# Sports anaemia and anthropometric evaluation of footballers at Kwame Nkrumah University of Science and Technology (KNUST)

**DOI:** 10.11604/pamj.2016.24.25.7244

**Published:** 2016-05-09

**Authors:** Clement Opoku-Okrah, Daniel Kwasi Sam, Bernard Nkum, Elliot Eli Dogbe, Lilian Antwi-Boateng, Benedict Sackey, Daniel Gyamfi, Kwabena Owusu Danquah

**Affiliations:** 1 Department of Medical Laboratory Technology, Faculty of Allied Health Sciences, Kwame Nkrumah University of Science and Technology, Kumasi, Ghana; 2 School of Medical Sciences, Kwame Nkrumah University of Science and Technology, Kumasi, Ghana; 3 Transfusion Medicine Unit, Komfo Anokye Teaching Hospital, Kumasi, Ghana

**Keywords:** Anthropometric, haematological parameters, footballers, Kumasi

## Abstract

**Introduction:**

Sports anaemia is a physiological activity that occurs amongst footballers and may be due to poor diet, over-training, as well as an increase in plasma volume in endurance training activities. High plasma volume leads to changes in haematological parameters that may impact on endurance of footballers. The objective of the study was to determine the correlation between haematological and an-thropometric indices and their role in sports anaemia in a tropical setting.

**Methods:**

Venous blood was taken into EDTA for 12 soccer players of KNUST soccer team before training and after training for the first (W1) and fifth (W5) weeks of training sessions. Complete blood count analysis was done for each blood sample and anthropometric parameters such as height, weight, body mass index, body fat percent and lean body mass were also measured. Cross-tabulations with mean and standard deviation or median and range were computed. Paired t-test & and Mann-Whitney test for parametric and non-parametric data computations were carried out and a p-value ≤ 0.05 was taken to rep-resent significant difference between data groups.

**Results:**

There was significant reduction in haemoglobin (p = 0.003), haematocrit (p = 0.002), mean cell volume (MCV) (p = 0.034) and red blood cell (RBC) count (p = 0.011) as a result of a significant expansion of plasma volume (p= 0.006). Neutrophil, lymphocyte and eosinophil counts were reduced significantly (p= 0.043, 0.001 and 0.007, respectively) after the training at W5. Lean body mass (LBM) inversely correlated with haemoglobin (r = -0.787, p = 0.002) and haematocrit (r = -0.588, p = 0.044). Body fat percentage (BFP) also negatively correlated with lymphocyte count (r = -0.700, p = 0.011). Furthermore, there was a positive correlation between body mass index (BMI) and plasma volume change after the training programme (r = 0.689, p = 0.013).

**Conclusion:**

The results suggest that sports anaemia was induced by an increase in plasma volume that resulted in changes in haematological parameters.

## Introduction

Soccer is a rigorous exercise, which needs a lot of energy. However, reports indicate a significant decrease in haematological parameters such as haemoglobin concentration, haematocrit and red blood cell counts, due to it being an endurance exercise [1, 2]. Such changes in these parameters can lead to a condition known as sports anaemia [3] or delusional pseudo-anaemia [4]. Anaemia is a deficiency of functional mature erythrocytes, which leads to a decreased oxygen delivery to the body tissues. This usually leads to clinical consequences such as weakness, fatigue, increased cardiac output and tissue hypoxia. Anaemia may be classified by levels of red blood cell production, destruction or loss, morphology, and haematological indices such as hae-moglobin concentration, mean cell volume (MCV), mean cell haemoglobin concentration (MCHC) and mean cell haemoglobin (MCH). [4, 5] reported that transient increase in plasma volume during exercise contributes to sports anaemia not attributable blood loss. A study by [6] observed that haematocrit fell from 43.5% to 37.9% after 5 days of hill walking. Blood volume changes are often seen as an important adaptation to perspiration imposed during physical exercise [5]. Red blood cell volume expansion often occurs slowly over many weeks to months, while plasma volume expansion, which happens rapidly, spans over hours to days [7]. Although, there is a consensus that alteration of plasma volume occurs during exercise, some other investigations have identified an absence of plasma volume expansion [5]. Acute plasma expansion reduces cardiovascular strain during exercise in high temperatures [5], providing a better endurance capability for athletes. A higher plasma volume can increase exercise capacity through an increased cardiac output and by reducing blood viscosity, thereby optimizing micro-circulation and improving oxygen delivery to the working muscle [2]. However, it can impact negatively since it can lead to significant reduction in haematological parameters such as haemoglobin concentration, haematocrit and red blood cell counts. It is known that haemoglobin concentration increases during exercise as a result of the activation of erythrocytosis; however an increase in plasma volume during exercise has been detected to cause an apparent shutdown of erythrocytosis [2]. A study conducted by Silva et al., 2008) showed an increase in erythrocytes, haemoglobin and haematocrit attributing the increase to the model of the training programme which consisted of 20 sessions with a mean volume of 14.66 hours/week between T1 (week 0) and T2 (week 6). Between T2 and T3 (week 12), the soccer players performed 24 sessions with a mean volume of 16.33hours/week.

Soccer is a sport that cannot only be classified as endurance and strength training [8]; it includes a series of runs and tackles, which involve much energy and aerobic metabolism. Even though fluid intake is important, practical challenge how to identify the specific soccer player and environmental conditions in which exercising soccer should be done as well as matching fluid intake with sweating rate [9]. Moreover, the eventual activation of thirst leads to water replacement, which may increase plasma volume and contribute to the phenomenon of sports anaemia. Aerobic metabolism stimulates mobilisation of erythrocytes into peripheral circulation due to increased energy requirement. Thus when athletes are anaemic, it results in physical under-performance. The effect of soccer has also been observed to have impact on leucocyte populations which in-creases during training [10–12]. The reason for the increase has been attributed to an inflammatory state that is induced during soccer training [13], as well as an increase in demargination of leucocytes [14]. Contrarily, [15], observed a decrease in WBC, RBC and platelets counts among elite female soccer players after both moderate and heavy training sections. Duzova et al. [16], observed an in-crease in neutrophil superoxide activity as well as a decrease in antioxidant activity during maximal exercise. The success of sports is also related to the anthropometry of the participants, which is a fundamental entity for competitive success. Physical performance can be defined as human body competence in strength, speed, endurance, agility and flexibility in playing sport [17]. Performance is related not only to anatomical and physiological characteristics, but also with training level and nutritional condition [17]. Different dietary and training strategies within a season elicit variable changes in body composition of athletes [18]. In rugby, for instance desirable changes in body composition (increases in lean mass and/or decreases in skin folds) occur primarily during prepa-ration for competition when training volume is high [19]. Some studies have established a correlation between sports and anthropometry of sportsmen. Banfi et al. [20] established a positive correlation between lean body mass and haemoglobin concentration in elite rugby players. A 10-week study involving young soccer players conducted by [21] revealed an increase in the anthropometric characteristics such as body mass index, body mass and lean body mass. However, there is little consensus whether the physical characteristics of athletes can influence the rate of plasma volume expansion and eventually lead to sports anaemia. This study seeks to determine the correlation between haematological and anthropometric values and their role in sports anaemia among footballers in a tropical setting.

## Methods

### Study population

Twelve Ghanaian male footballers (age range, 18-24years) Kwame Nkrumah University of Science and Technology (KNUST) football team who were being prepared for the Mini Ghana University Sports Association (GUSA) games were recruited in the study from January to April, 2013. Recruitment and sampling was at Paa Joe stadium at KNUST. The selected footballers were informed of all the procedures involved in this study. Ethical approval for the study was obtained from the Committee on Human Research, Publications and Ethics (CHRPE) of KNUST.

### Inclusion and exclusion criteria

The study recruited apparently well footballers deemed fit to carry out the coach’s scheduled training exercises. Unwell and injured footballers of the team were excluded.

### Sampling procedures

Subjects were evaluated at two different times along the study. The sampling periods were designated as W1 (week 1) and W2 (week 5), denoting the pre-training and the ending of the training programme. For W1 and W2, blood samples were collected in the afternoon at 4:00pm, be-fore the onset of training and after the training programme. The footballers were made to undergo anthropometric measurements on the next day before their training.

### Training Programme

The coach of the team designed the training programme. Table 1 describes the content of the training programme specific to each week of training.

**Table 1:** Soccer training programme from week 1(W1) to week 5(W2)

Specific Training	Week 1	Week 2	Week 3	Week 4	Week 5
Endurance	4	3	3	3	
Flexibility	5	3	3	3	
Skills and tactical	3	4	6	6	Simulated Matches
Simulated game	2	4	4	4	
Strength training	0	2	5	3	

All training sessions were preceded by 10 minutes of warm-up consisting of half field sprint, line touches and muscle stretching aerobics. Numbers in parenthesis represents weekly frequency

### Blood Analysis

Venous blood sample (2.5ml) was obtained using standard phlebotomy protocol into vacutainer tubes containing K3EDTA. Blood samples were transported to Komfo Anokye Teaching Hospital (KATH) in a cold box to prevent deterioration of blood with time if the one-hour allowance is exceeded. Haematological analysis was performed using Sysmex XT 4000i haematology analyser (Sysmex Corporation, Kobe-Japan) at the Haematology Department of KATH. Standard quality control protocol for the automated haematology analyser, which, requires passing three levels (normal, low and high) of assessment with Sysmex quality control samples was ensured before test samples were analysed. The haematological parameters which were measured include red blood cell (RBC), haemoglobin, haematocrit, mean cell volume (MCV), mean cell haemoglobin (MCH), mean cell haemoglobin concentration (MCHC), white blood cell (WBC), mean platelet volume (MPV), differential count (neutrophils, eosinophils, basophils, monocytes and lymphocytes) and platelet count. Percentage changes in plasma volume were calculated using the method described by Dill and Costill [22].

### Anthropometric evaluation

The height of participants were measured with a Seca stadiometer to the nearest 0.1cm whiles the weight was measured with a Salter weighing scale to the nearest of 0.5kg. Anthropometric measurements determined include: height (m), weight (kg), body mass index [BMI (kg/m^2^) = weight/height^2^], body fat percentage (BFP,%), and lean body mass (LBM, kg). Body fat percentage was estimated using the formula developed by Hodgdon et al., [23].

### Statistical analysis

Results obtained were stored in Microsoft Office Excel 2007 and statistically analysed using Statistical Package for Social Sciences (SPSS for Windows, Version 16.0. Chicago, SPSS Inc.). The normality of the data was assessed using Shapiro-Wilk statistic. Paired t-test was used to com-pare difference of means before and after training. Non-parametric test (Mann-Whitney test) was used to compare the difference of means between W1 and W2. Correlations between the parameters were performed using the Pearson's correlation. All data obtained from the study were ex-pressed as mean ± standard deviation or median with inter-quartile ranges. A significance level of p ≤ 0.05 was used.

### Anthropometric characteristics of footballers between W1 and W2

The weight, height, body mass index (BMI), body fat percentage (BFP) and lean body mass (LBM) of the twelve footballers sampled during the training programme are shown in Table 2. Anthropometric characteristics showed insignificant variations (p > 0.05) between the first week of training (W1) and the fifth week of training (W2).

**Table 2:** Anthropometric characteristics of football players during the soccer training programme

Parameters	W1 (mean ± SD)	W2 (mean ± SD)	p-value
Weight (kg)	70.67 ± 6.33	71.00 ± 6.27	0.898
Height (m)	1.75 ± 0.07	1.75 ± 0.07	0.975
BMI (kg/m^2^)	23.10 ± 1.72	23.23 ± 1.71	0.855
BFP (%)	9.13 ± 2.57	9.42 ± 1.95	0.755
LBM (kg)	64.18 ± 5.56	64.26 ± 5.12	0.973

BMI refers body mass index; BFP refers to body fat percentage

LBM refers to lean body mass; W1 is week 1 of training programme W2 is week 5 of training programme

### Haematological parameters of footballers at W1 and W2

At the end of week 1 (W1), haematological parameters showed insignificant changes (p > 0.05) from the pre-training values except for mean cell volume (MCV) which showed significant reduction after training (Table 3). At W2, there was a significant reduction in red blood cell (RBC) count, haemoglobin concentration and haematocrit due to loss of plasma volume after training as shown in Table 3. Progressive compensatory adaptation resulted in a significant (p = 0.009) plasma volume gain at the end of the fifth week (W2) training session compared to that of week one (W1). Accompanied with this is a dilution of peripheral RBC count (p = 0.004) and consequently, haemoglobin concentration (p = 0.003) and haematocrit (p = 009) compared to the pre-training levels of these parameters on the fifth week. RBC count at the end of training session for week one was not significantly different (p = 0.244) from that the value before the training however, at the end of week five training session the RBC count was significantly lower (p= 0.011) than that at the end of week one training. Haemoglobin and haematocrit levels before week five training session were considerably lower (p = 0.048 and 0.029 respectively) compared to that before week one training. Again, Haemoglobin and haematocrit levels at the end of week five training session were significantly lower (p = 0.003 and 0.002 respectively) to that at the end of week one training. There was a significant (p = 0.001) increase between the pre and post training neutrophil count at the end of the W1 but this did not influence significantly (p = 0.083) the overall total leukocyte count. Eosinophil count decreased persistently from W1 through W2 after training while lymphocytes count also decreased at W2. Neutrophil count showed a significant decrease (p = 0.043) be-tween post-training measurements of W1 and W2 (Table 4). Correlation between haematological and anthropometric parameters showed lean body mass (LBM) correlating negatively with haemoglobin and haematocrit (Table 5). The study also observed positive correlation between changes in plasma volume at W1 and body mass index (BMI) (r = 0.689; p-value = 0.011). Weight and height also negatively correlated with haemoglobin, while body fat percentage negatively correlated with lymphocyte count at W1 (Table 5).

**Table 3:** Comparison of erythrocyte parameters and changes in plasma volume of football players between W1 (week 1) and W2 (week 5)

	W1			W2		
Parameters	Before Training	After Training	p-value	Before Training	After Training	p-value
RBC (X10^12^/L)	5.22 ± 0.32	5.25 ± 0.33	0.244	5.01 ± 0.31	4.90 ± 0.29#	0.004
Haemoglobin (g/dL)	14.66 ± 0.64	14.77 ± 0.68	0.285	14.08 ± 0.72*^1^	13.81 ± 0.73§^1^	0.003
Haematocrit (%)	41.54 ± 1.70	41.61 ± 1.84	0.760	39.82 ± 1.90*^2^	39.01 ± 1.76§^2^	0.009
MCV (fL)	79.82 ± 4.18	79.40 ± 4.07	0.034	79.69 ± 4.15	79.78 ± 4.04	0.740
MCH (pg)	28.17 ± 1.60	28.18 ± 1.56	0.886	28.18 ± 1.61	28.24 ± 1.57	0.339
MCHC (g/dL)	35.29 ± 0.84	35.50 ± 1.09	0.111	35.34 ± 0.83	35.39 ± 0.77	0.690
Δ in Plasma Volume	-1.870 (-3.369-1.659)			2.159¶(0.588 - 5.300)		

# Statistical difference between W1 and W2 (after training) with p-value of 0.011

*1,2 Statistical difference between W1 and W2 (before training) with p-value of 0.048 and 0.029 respectively

§^1,2^ Statistical difference between W1 and W2 (after training) with p-value of 0.003 and 0.002 respectively

¶ Statistical difference between W1 and W2 with a p-value of 0.009; RBC refers to red blood cell; MCV refers to mean cell volume; MCH refers to mean cell haemoglobin; MCHC refers to mean cell haemoglobin concentration.

Δ refers to percentage median change in plasma volume

**Table 4:** Characteristics of leucocyte and platelet parameters of football players between W1 (week 1) and W2 (week 5)

	W1			W2		
Parameters	Before Training	After Training	p-value	Before Training	After Training	p-value
WBC (x10^3^/µL)	6.68 ± 1.70	7.14 ± 1.93	0.083	6.16 ± 1.57	5.85 ± 1.51	0.229
Neutrophils (x10^3^/µL)	2.81 ± 0.98	3.89 ± 1.56	0.001	2.39 ± 0.96	2.73 ± 1.01§	0.123
Eosinophils (x10^3^/µL)	0.24 ± 0.17	0.21 ± 0.18	0.003	0.25 ± 0.16	0.18 ± 0.11	0.007
Basophils (x10^3^/µL)	0.06 ± 0.05	0.09 ± 0.08	0.352	0.08 ± 0.03	0.08 ± 0.07	0.926
Lymphocytes (x10^3^/µL)	2.73 ± 1.20	2.36 ± 0.52	0.206	2.90 ± 0.72	2.37 ± 0.69	0.001
Monocytes (x10^3^/µL)	0.59 ± 0.22	0.60 ± 0.22	0.676	0.54 ± 0.23	0.50 ± 0.18	0.295
Platelets (x10^3^/µL)	218.25 ± 54.10	243.58 ± 57.26	0.146	221.92 ± 64.61	244.92 ± 59.58	0.096
MPV (fL)	10.39 ± 0.73	10.20 ± 0.75	0.317	10.20 ± 0.63	10.07 ± 0.83	0.406

§ Significant difference between W1(after training) and W2 (after training) with a p-value of 0.043

WBC refers to white blood cell; MPV refers to mean platelet volume

**Table 5:** Pearson correlation between anthropometric characteristics and erythrocyte parameters of soccer players at W1 (week 1)

	Weight	Height	BMI	BFP	LBM
B RBC	-0.509	-0.553	-0.023	-0.011	-0.509
A RBC	-0.617*	-0.474	-0.241	-0.117	-0.585*
B Haemoglobin	-0.657*	-0.802**	0.053	-0.105	-0.635*
A Haemoglobin	-0.852**	-0.724**	-0.273	-0.246	-0.787**
B Haematocrit	-0.589*	-0.654*	-0.043	-0.068	-0.582*
A Haematocrit	-0.658*	-0.475	-0.319	-0.260	-0.588*
B MCV	0.156	0.140	0.012	-0.015	0.153
A MCV	0.214	0.181	0.041	-0.053	0.226
B MCH	0.052	-0.024	0.078	-0.052	0.064
A MCH	0.001	-0.071	0.066	-0.046	0.011
B MCHC	-0.213	-0.372	0.174	-0.052	-0.193
A MCHC	-0.351	-0.425	0.051	-0.002	-0.352
Plasma change	0.413	-0.166	0.689*	0.384	0.294

* Correlation is significant at the 0.05 level (2-tailed)

** Correlation is significant at the 0.01 level (2-tailed)

Values have been expressed as correlation coefficient; “A” refers to after training and “B” refers to before training

## Results

### Anthropometric characteristics of footballers between W1 and W2

The weight, height, body mass index (BMI), body fat percentage (BFP) and lean body mass (LBM) of the twelve footballers sampled during the training programme are shown in Table 2. Anthropometric characteristics showed insignificant variations (p > 0.05) between the first week of training (W1) and the fifth week of training (W2).

### Haematological parameters of footballers at W1 and W2

At the end of week 1 (W1), haematological parameters showed insignificant changes (p > 0.05) from the pre-training values except for mean cell volume (MCV) which showed significant reduction after training (Table 3). At W2, there was a significant reduction in red blood cell (RBC) count, haemoglobin concentration and haematocrit due to loss of plasma volume after training as shown in Table 3. Progressive compensatory adaptation resulted in a significant (p = 0.009) plasma volume gain at the end of the fifth week (W2) training session compared to that of week one (W1). Accompanied with this is a dilution of peripheral RBC count (p = 0.004) and consequently, haemoglobin concentration (p = 0.003) and haematocrit (p = 009) compared to the pre-training levels of these parameters on the fifth week. RBC count at the end of training session for week one was not significantly different (p = 0.244) from that the value before the training however, at the end of week five training session the RBC count was significantly lower (p= 0.011) than that at the end of week one training. Haemoglobin and haematocrit levels before week five training session were considerably lower (p = 0.048 and 0.029 respectively) compared to that before week one training. Again, Haemoglobin and haematocrit levels at the end of week five training session were significantly lower (p = 0.003 and 0.002 respectively) to that at the end of week one training. There was a significant (p = 0.001) increase between the pre and post training neutrophil count at the end of the W1 but this did not influence significantly (p = 0.083) the overall total leukocyte count. Eosinophil count decreased persistently from W1 through W2 after training while lymphocytes count also decreased at W2. Neutrophil count showed a significant decrease (p = 0.043) be-tween post-training measurements of W1 and W2 (Table 4). Correlation between haematological and anthropometric parameters showed lean body mass (LBM) correlating negatively with haemoglobin and haematocrit (Table 5). The study also observed positive correlation between changes in plasma volume at W1 and body mass index (BMI) (r = 0.689; p-value = 0.011). Weight and height also negatively correlated with haemoglobin, while body fat percentage negatively correlated with lymphocyte count at W1 (Table 5)

## Discussion

It is often expected in endurance training that, due to increased metabolic activities, erythrocytes will be pooled into circulation to aid in endurance. However, this is often not achieved due to poor diet, over-training, and possibly due to a transient increase in plasma volume. Sports anaemia creates the impression of low haemoglobin in athletes. This study investigated the impact of plasma dilution as a result of endurance training on haematological parameters and the possible correlation between anthropometry of footballers and haematological parameters.

### Anthropometric characteristics of soccer players

**Figure 1:**
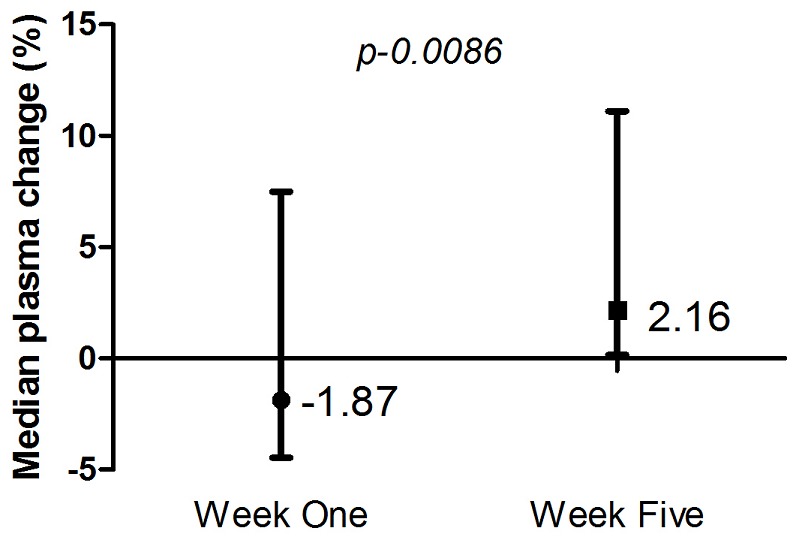
Variation of plasma volume between training periods (W1, W2)

This study did not record any significant changes in the anthropometric characteristics of the footballers (Table 2) between the first week of training (W1) and the fifth week (W5) of training. This seems contrary to observed significant increase in body mass, body mass index and lean body mass observed by Miranda et al., [21] in a 10 week training session of youth football players in Brazil. The difference in the observation may be attributed to the longer duration of training sessions (10 weeks) used in their study. From Table 5, the lean body mass (LBM) of the soccer players negatively correlated with haemoglobin (r = -0.787) and haematocrit (r = -0.588), at W1 where the high loss of plasma volume was recorded (Figure 1) and less strongly at W2 (Table 6) where rather increase in plasma volume occurred. This observation contrasts that reported among elite rugby players during competitive season by Banfi et al [20], who found a positive correla-tion between LBM and haemoglobin. Weight and height also negatively correlated with haemo-globin. The study observed a positive correlation between changes in plasma volume at W1 and body mass index (BMI) at W1. This may be explained by the observed relationship between high BMI and hyperhidrosis [24], a pathological condition, which is associated with excessive sweating. Even though sports-related studies have not looked at the impact of BMI on sweating, and on plasma volume expansion, it may be suggested that the BMI of the footballers might have led to the increase in plasma volume. With respect to the leucocyte parameters, BFP was found to negatively correlate with lympho-cyte count at W1. Nieman et al. (1999) found that, as BFP increases, leucocytes (including neu-trophils and lymphocytes) also increase. In their observation, Tsubakihara et al. [12] indicated that training activities increase the presence of oxygen free radicals which can induce lipid per-oxidation and increase the apoptosis of lymphocytes [25] which could lead to a reduction in lymphocyte count after training as seen in this study. This finding is clinically relevant since, many footballers train without considering the impact of the training on their health. Strenuous exercise might therefore increase the susceptibility of one to infections as a result of the decrease in lymphocyte count.

**Table 6:** Pearson correlation between anthropometric characteristics and erythrocyte parameters of soccer players at week five (W2)

	Weight	Height	BMI	BFP	LBM
B RBC	-0.462	-0.409	-0.140	-0.515	-0.365
A RBC	-0.409	-0.475	-0.004	-0.366	-0.347
B Haemoglobin	-0.522	-0.660*	0.027	-0.249	-0.513
A Haemoglobin	-0.389	-0.702*	0.232	-0.055	-0.421
B Haematocrit	-0.430	-0.472	-0.062	-0.327	-0.390
A Haematocrit	-0.303	-0.497	0.112	-0.098	-0.317
B MCV	0.158	0.046	0.120	0.327	0.076
A MCV	0.210	0.104	0.114	0.360	0.120
B MCH	0.039	-0.155	0.190	0.343	-0.060
A MCH	0.063	-0.180	0.240	0.363	-0.040
B MCHC	-0.263	-0.498	0.208	0.120	-0.324
A MCHC	-0.347	-0.729**	0.354	0.077	-0.398
Plasma change	-0.329	0.111	-0.509	-0.564	-0.202

* Correlation is significant at the 0.05 level (2-tailed)

** Correlation is significant at the 0.01 level (2-tailed)

Values have been expressed as correlation coefficient; “A” represent after training and “B” refers to before training.

### Effects of soccer training on haematological profile

This study identified the impact of the soccer-training programme on haematological parameters. The significant increase (p = 0.0086) in plasma volume at W2 (week 5) (Figure 1) collaborated with the work done by Sawka et al. [5] and Karakoc et al. [26]. This phenomenon of plasma vol-ume expansion is to compensate for the negative effects of acute exercise-induced haemoconcen-tration [2], which is caused by increased levels of aldosterone and osmotically active plasma pro-teins as well as a decrease in urodilatin activity. These mechanisms eventually lead to fluid reten-tion and thus the increase in plasma volume [1, 27]. The study observed a significant reduction in haemoglobin, red blood cell (RBC) and haema-tocrit between W1 and W2, which might be due to the significant increase in plasma volume dur-ing the training programme (Table 3). The period before and after training at W1, showed a sig-nificant decrease in mean cell volume (MCV) (p= 0.034), which agrees with observations by others [8, 26, 28] as explained by the expansion of the plasma volume leading to haemodilution. Neutrophil and leucocyte counts were found to increase during athletic training exercises [10, 11, 29]. At W1, there was a significant increase (p = 0.001) in neutrophil count after training. These changes might be due to the extent of the training exercise, as well as an inflammatory state that is induced during exercise [13]. The increase in neutrophil count is explained by the fact that muscles tend to disintegrate because they become weak leading to the release of in-flammatory cytokines, growth hormones and stress hormones [13]. It is reported that the release of inflammatory cytokines leads to the release of adrenocorticotrophic hormone (ACTH), which stimulates the release of cortisol from the adrenal gland and thereby, increases leucocyte count, as a physiological response to the inflammatory state [13]. Pedersen et al. [13] had suggested that an increase in neutrophil is affected by an increase in plasma levels of cortisol, noradrenaline and adrenaline. Increase in neutrophils may also be explained by the process of demargination [14], where loosely bound leucocytes get dislodged upon exercising, thereby leading to an increase in leucocyte count. The present study also observed a decrease in neutrophil between W1 and W2, which contrasts observations by other researchers [12, 13, 30], but was in agreement with the study of Fallon et al. (2001) who observed a significant decrease in neutrophil count in women after heavy soccer training. Our subjects were however all males. There was also a decrease in lymphocyte and eosinophil count after the training programme (Table 4). The decrease in lymphocyte count is in accordance with the observations of Tsubakihara et al. [12], and has been attributed to the in-crease in stress hormones and the impact of oxidative stress. It has been suggested that, reactive oxygen species (ROS) increase during repeated bouts of exercise [12, 16, 31] and thus reduce lymphocyte function and number as a result of increased lymphocyte apoptosis during and after training [25]. Eosinopenia, which occurred after the training programme, may be attributed to the effect from oxidative stress, as it happens for lymphopenia [32]. The clinical importance of these results to footballers will be the need to control the level of free radicals in circulation after train-ing, since they can lead to a reduction in immunity and contribute to infections and oxidative stress. Some researchers have concluded that platelet count increases after exercise [26, 33, 34] and this has been attributed to exercise-induced thrombocytosis, however, this study did not record a sig-nificant increase between W1 and W2 (p= 0.956).

## Conclusion

This study observed an increase in plasma volume, which correlated with a decrease in red blood cell count, haemoglobin, mean cell volume and haematocrit. Correlations between anthropometry of the footballers and haematological parameters suggest the possible influence of anthropometric characteristics on sports performance and haematological parameters. There was also significant reduction in eosinophil, lymphocyte and neutrophil counts. All these results are very critical to the performance as well as the immunity of footballers. It is noteworthy, that athletes in general are made aware of the consequences of overtraining and/or strenuous training on their immune system and their performance. It is therefore recommended that further studies be carried out using a similar training programme to evaluate the effect of exercise on immunity and the correlation between sports anaemia and performance of footballers.

### What is known about this topic

- Sport anaemia is a common physiological condition in which an increase in plasma volume instead of blood loss creates an artificial reduction in RBCs. It has been reported in hill climbers and soccer players.
- There is an increase in the concentration of RBC due to excessive loss of water in sweat. This is compensated for by increase in plasma volume leading to artificial or pseudo anaemia.

### What this study adds

- We demonstrated for the first time that there was a decrease in neutrophil count among male footballers.
- We also found a decrease in eosinophils at the end of week five (5) of training.
